# Personalized screening intervals for kidney function in patients with chronic heart failure: a modeling study

**DOI:** 10.1007/s40620-021-01014-0

**Published:** 2021-03-18

**Authors:** Anne-Sophie Schuurman, Anirudh Tomer, K. Martijn Akkerhuis, Ewout J. Hoorn, Jasper J. Brugts, Olivier C. Manintveld, Jan van Ramshorst, Victor A. Umans, Eric Boersma, Dimitris Rizopoulos, Isabella Kardys

**Affiliations:** 1 Department of Cardiology, Erasmus MC University Medical Center, Room Na-316, ‘s Gravendijkwal 230, 3015 CE Rotterdam, The Netherlands; 2 Cardiovascular Research School COEUR, Erasmus MC University Medical Center, Rotterdam, The Netherlands; 3 Department of Biostatistics, Erasmus MC University Medical Center, Rotterdam, The Netherlands; 4 Department of Internal Medicine, Erasmus MC University Medical Center, Rotterdam, The Netherlands; 5 Department of Cardiology, Northwest Clinics, Alkmaar, The Netherlands

**Keywords:** Chronic heart failure, Kidney biomarkers, Risk assessment, Personalized, Screening

## Abstract

**Background:**

High mortality and rehospitalization rates demonstrate that improving risk assessment in heart failure patients remains challenging. Individual temporal evolution of kidney biomarkers is associated with poor clinical outcome in these patients and hence may carry the potential to move towards a personalized screening approach.

**Methods:**

In 263 chronic heart failure patients included in the prospective Bio-SHiFT cohort study, glomerular and tubular biomarker measurements were serially obtained according to a pre-scheduled, fixed trimonthly scheme. The primary endpoint (PE) comprised cardiac death, cardiac transplantation, left ventricular assist device implantation or heart failure hospitalization. Personalized scheduling of glomerular and tubular biomarker measurements was compared to fixed scheduling in individual patients by means of a simulation study, based on clinical characteristics of the Bio-SHiFT study. For this purpose, repeated biomarker measurements and the PE were jointly modeled. For personalized scheduling, using this fitted joint model, we determined the optimal time point of the next measurement based on the patient’s individual risk profile as estimated by the joint model and the maximum information gain on the patient’s prognosis. We compared the schedule’s capability of enabling timely intervention before the occurrence of the PE and number of measurements needed.

**Results:**

As compared to a pre-defined trimonthly scheduling approach, personalized scheduling of glomerular and tubular biomarker measurements showed similar performance with regard to prognostication, but required a median of 0.4–2.7 fewer measurements per year.

**Conclusion:**

Personalized scheduling is expected to reduce the number of patient visits and healthcare costs. Thus, it may contribute to efficient monitoring of chronic heart failure patients and could provide novel opportunities for timely adaptation of treatment.

**Graphic abstract:**

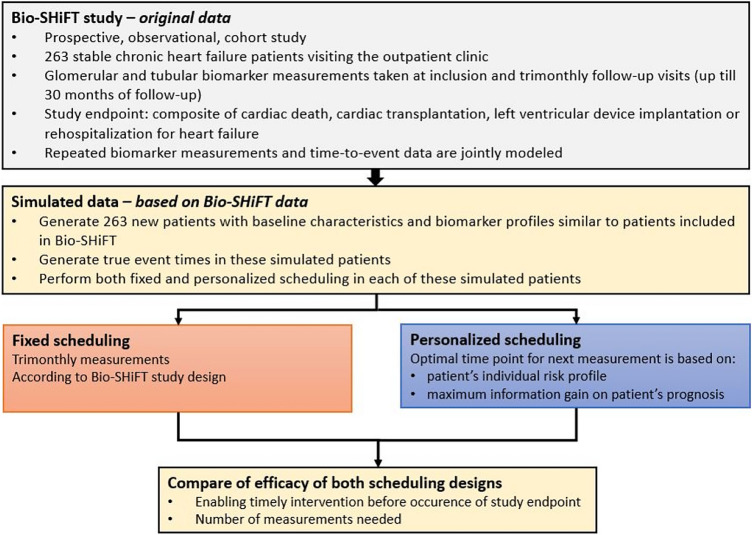

**Supplementary Information:**

The online version contains supplementary material available at 10.1007/s40620-021-01014-0.

## Introduction

Since reduced kidney function is associated with poor prognosis in patients with chronic heart failure (CHF), [1] guidelines recommend monitoring kidney function [2]. However, monitoring frequency has not yet been scientifically substantiated. In the Bio-SHiFT study, we demonstrated that individual temporal evolution of kidney biomarkers is associated with poor outcome in CHF patients [3]. Moreover, we demonstrated a method to derive personalized, dynamic risk estimates for adverse outcome based on such temporal biomarker evolutions in individual CHF patients [3]. These estimates are updated after every measurement, providing incremental information on the patient’s prognosis [4].

Such personalized dynamic risk assessments could potentially also be used to derive personalized screening intervals for individual CHF patients. Thus, timing of the patient’s next outpatient visit could be tailored to their individual biomarker evolution so far [5]. Personalized screening intervals aim to maximize information gain on the individual patient’s disease progression, while minimizing the number of necessary measurements [5]. Using data of the Bio-SHiFT study, we recently demonstrated that personalized scheduling of N-terminal pro-B-type natriuretic peptide (NT-proBNP) measurements, as compared to a pre-defined trimonthly fixed scheduling approach, shows similar prognostic performance but requires fewer NT-proBNP measurements [6].

The individual temporal patterns we examined in the Bio-SHiFT study that were associated with adverse cardiac outcome included glomerular function markers [creatinine, cystatinC and estimated Glomerular Filtration Rate (eGFR)], tubular markers [N-acetyl-beta-d-glucosaminidase (NAG), and the kidney injury molecule (KIM)-1] [3]. Moreover, we found a similar association with adverse outcome for high-sensitive troponin T (HsTNT) [7]. In the current study, using Bio-SHiFT data, we aim to compare personalized scheduling of these biomarkers to a pre-defined fixed scheduling approach in individual CHF patients.

### Short methods

In 263 stable CHF patients from the Bio-SHiFT study who underwent trimonthly sampling, we measured glomerular function [creatinine, cystatinC, eGFR_creat_, eGFR_cysC_ (both based on CKD-EPI equation)] in a total of 1984 plasma samples [median: 9 (25th–75th percentile: 5–10) per patient] and assessed tubular function (NAG, KIM-1) in 1912 urine samples [median: 8 (25th–75th percentile: 5–10) per patient] [3]. The primary endpoint (PE) was defined as the composite of cardiac death, cardiac transplantation, left ventricular assist device implantation or heart failure hospitalization, whichever occurred first. Using joint models for time-to-event and longitudinal data, we modeled the association between repeated kidney biomarker measurements and the PE [3, 8]. After adjustment for age, sex, diabetes, atrial fibrillation, NYHA class, diuretics, systolic blood pressure, and eGFR_creat_ (for tubular markers), we obtained a hazard ratio (HR) with 95% confidence interval (CI) that estimated the risk of the PE associated with a 20% increase or decrease in biomarker level.

Measurements in the Bio-SHiFT study were obtained according to a pre-scheduled, fixed trimonthly scheme. In the Bio-SHiFT study, there was no study arm that applied a personalized screening procedure, and thus direct assessment of personalized scheduling was not possible. Therefore, we performed a simulation study in which we could compare the performance of the personalized versus the fixed schedule. First, we generated 263 patients with baseline characteristics and biomarker profiles similar to the original Bio-SHiFT population [5]. Based on this, we fitted a new joint model for these 263 patients. By using the latter model, we compared scheduling of biomarker measurements according to a pre-defined, fixed (trimonthly) screening design and a personalized screening design in these patients.

**Fig. 1:**
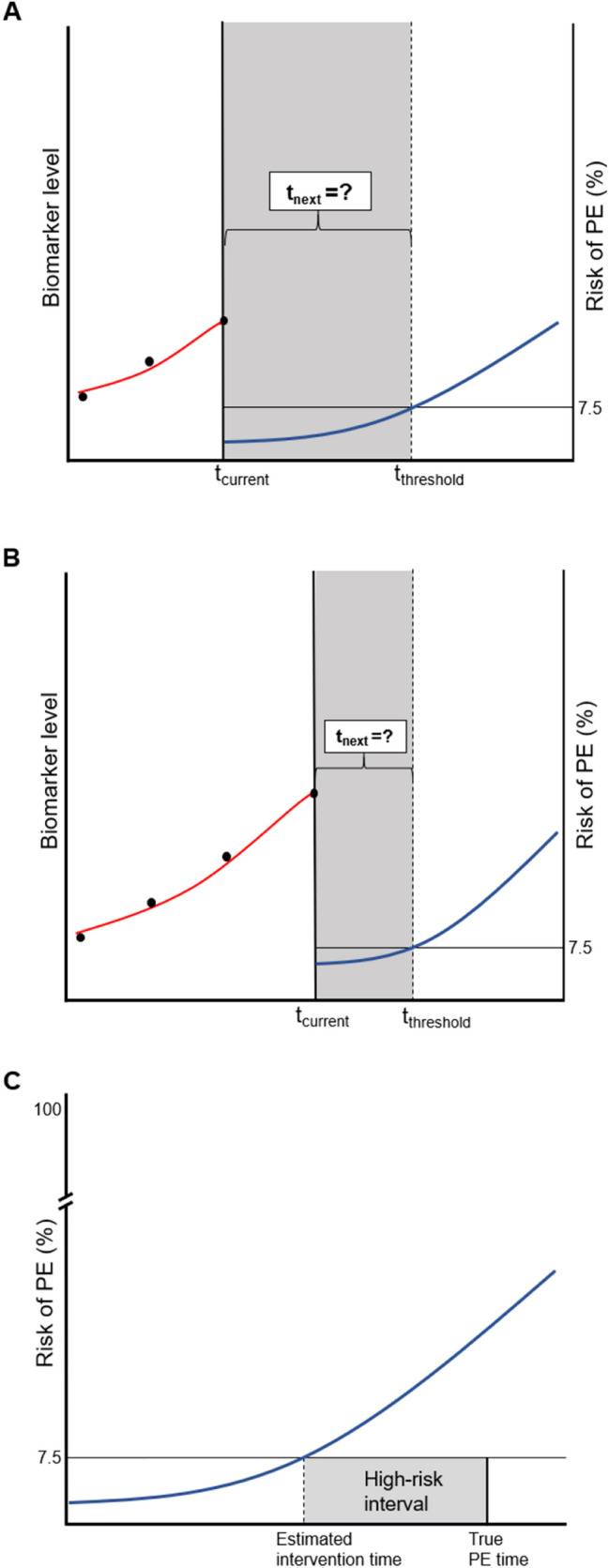
Illustration of personalized scheduling of biomarker measurements. **a** Example of a patient with three serially measured biomarker levels (dots) available until the current visit (*t*_current_). A personalized risk profile is derived using these three serially measured biomarker levels. In particular, the fitted joint model is used to find the time point at which the patient’s cumulative risk of PE (blue curve) is 7.5% (*t*_threshold_). The next biomarker measurement (*t*_next_) will be scheduled between the current visit and this time point. Subsequently, we use the fitted joint model to estimate the expected information gain on the patient’s prognosis at every time point within this specified time window. Then, based on the Kullback-Leibler divergence, we schedule the next biomarker measurement at the optimal time point at which we expect the maximum information gain on the patient’s prognosis. **b** After this additional fourth biomarker measurement is performed in the patient, the personalized cumulative risk of PE is updated. Based on this updated personalized cumulative risk of PE, again, the time point at which the cumulative risk of PE is 7.5%, is determined. If the personalized cumulative risk of PE within 3 months is less than 7.5%, we proceed to schedule the next biomarker measurement. However, if the personalized cumulative risk of PE within the next 3 months exceeds 7.5%, scheduling is stopped in order to adjust therapy and avoid the imminent PE. **c** Definition of high-risk interval as used in the personalized scheduling approach. The ‘true PE time’ is generated by the simulation study. Based on the estimated biomarker profile, the patient’s risk of PE (%) is estimated by the personalized scheduling approach (curve). The time point at which this risk of PE exceeds the risk threshold is defined as the ‘estimated intervention time’. The start of the high-risk interval is defined as the estimated intervention time minus the true PE time (in months).

In the personalized screening design, in each patient at each follow-up visit, the fitted joint model was used to find the time point at which the patients’ cumulative risk of PE was 7.5% (arbitrarily chosen threshold). The next biomarker measurement was scheduled between the current visit and this time point. Subsequently, we used the fitted joint model to estimate the expected information gain on the patient’s prognosis at every time point within this specified time window. Then, based on the Kullback-Leibler divergence, we scheduled the next biomarker measurement at the optimal time point at which we expect the maximum information gain on the patient’s prognosis (Fig. 1a, b) [5]. After this additional biomarker measurement was performed in the patient, the personalized cumulative risk of PE was updated. Based on this updated personalized cumulative risk of PE, again, the time point at which the cumulative risk of PE was 7.5%, was determined. If the personalized cumulative risk of PE within 3 months was less than 7.5%, we scheduled the next biomarker measurement in this way. However, if the personalized cumulative risk of PE exceeded 7.5% within 3 months of a screening visit, scheduling was stopped in order to allow for a (potential) timely intervention and avoid the imminent PE [5]. We compared personalized scheduling with fixed trimonthly scheduling in terms of capability of identifying the start of high-risk intervals (i.e., whether timely intervention was possible before the occurrence of PE) and number of measurements needed (Fig. 1c).

## Results

### Baseline characteristics

Baseline characteristics and biomarker levels of the 263 patients who were included in the Bio-SHiFT study are shown in Table S1. Briefly, the mean (standard deviation) age of the Bio-SHiFT patients was 67 (13) years and 72% were men. Baseline characteristics of a simulated dataset were similar (Table S2) and no significant differences were found in the median values of the first three biomarker measurements in the original and simulated datasets (Table S3). In original Bio-SHiFT data, the PE occurred in 70 (26.6%) patients during a median (25th–75th percentile) follow-up of 2.2 (1.4–2.5) years. In the simulated dataset, since we used the first three biomarker measurements as a run-in period for the joint model, maximum follow-up duration was somewhat longer (specifically, we set the maximum at 3 years, instead of 2.5 years as in Bio-SHiFT). For example, for the creatinine dataset, median (25th–75th percentile) follow-up duration was 3.0 (1.9–3.0) years. Associations of temporal biomarker patterns with the PE have previously been described in detail [3], and are provided in Table S4 (original cohort and a simulated dataset).

### Personalized screening versus fixed schedule: high-risk interval and number of measurements

**Fig. 2:**
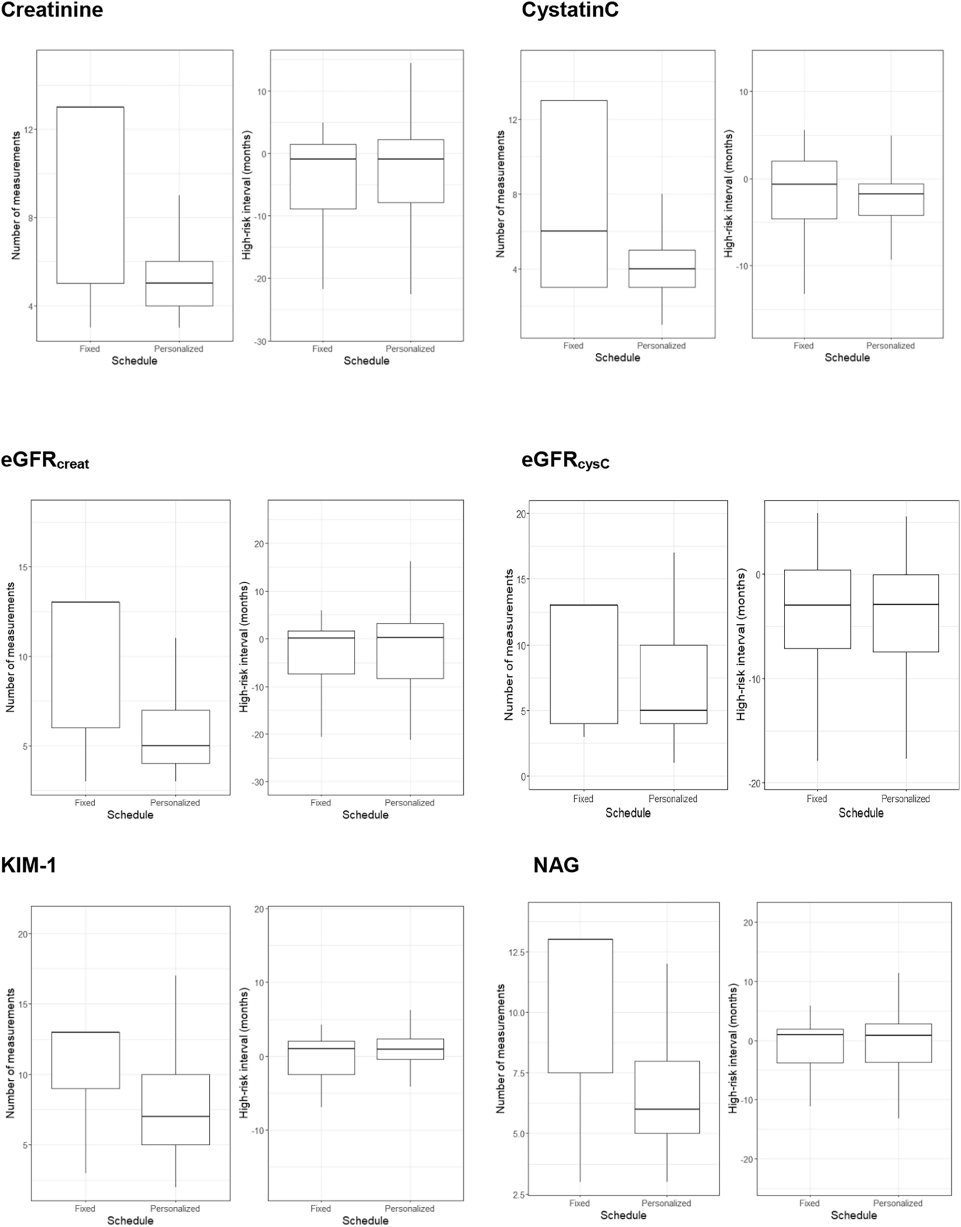
Comparison of personalized and fixed scheduling of kidney biomarkers.

The simulation study demonstrated that personalized scheduling required fewer glomerular biomarker measurements as compared to predefined, trimonthly fixed scheduling (Fig. 2). Specifically, over the full follow-up period, personalized scheduling of creatinine required a median (25th–75th percentile) of 5 (4–6), while fixed scheduling required a median of 13 (5–13) measurements. In other words, personalized scheduling of creatinine saved a median of 2.0 (0.0–2.7) measurements per year. Personalized scheduling for cystatinC also required a median (25th–75th percentile) of 2.7 (0.0–5.0) fewer measurements per year as compared to fixed scheduling [cystatinC, overall, personalized: median of 4 (25th–75th percentile: 3–5) and fixed: median of 6 [25th–75th percentile: 3–13)]. As demonstrated in Fig. 2, personalized scheduling of eGFR based on creatinine and cystatin C yielded similar results, and required a median (25th–75th percentile) of 2.7 (0.0–5.0) and 0.4 (0.0–3.4) fewer measurements per year, respectively. The same was true for tubular biomarker measurements KIM-1 [required median (25th–75th percentile) of 1.3 (0.0–2.2) fewer measurements per year] and NAG [median (25th–75th percentile): 1.5 (0.5–2.3) fewer measurements per year] (Fig. 2).

The performance of personalized and fixed scheduling regarding identification of the start of the high-risk interval (whether scheduling was stopped to enable treatment adaptation since the patient was at high-risk of the PE; and thus whether timely intervention was possible before the occurrence of PE) was similar, both for creatinine and cystatinC, as well as eGFR_creat_ and eGFR_cysC_ (Fig. 2). The same was true for the tubular biomarkers KIM-1 and NAG (Fig. 2).The result of personalized scheduling of HsTNT is presented in the Supplementary Materials.

## Discussion

We demonstrate that personalized scheduling of glomerular and tubular biomarkers in patients with CHF, as compared to fixed scheduling, requires fewer measurements per year, while performance regarding prediction of adverse cardiac events is similar. The frequency we used for the fixed schedule, i.e. trimonthly sampling, was chosen based on our study protocol, thus subjectively. Notably, even though our fixed schedules consisted of these rather frequent (trimonthly) biomarker measurements, the high-risk intervals identified by the personalized schedules were still similar, illustrating the value of the personalized approach. Since personalized scheduling required fewer measurements, this approach is expected to improve patient-relevant outcomes (fewer hospital visits and blood draws) as well as reduce healthcare costs compared to fixed scheduling. A further illustration on the practical application of the personalized screening approach to individual patients, with an elaborate individual example, is given in the Supplementary Material, Table S5–S6.

Study limitations include the use of only one testing set, and arbitrary assumptions that were made when developing the model and defining the risk threshold and risk window. Validation in larger studies is warranted. Of further note, we simulated the data for both the fixed and the personalized screening design and we did so to obtain a ‘fair’ comparison. In the original Bio-SHiFT data, pre-defined fixed trimonthly sampling was based on an underlying natural longitudinal process in the individual patients, which remains unknown. Since we do not have real data available on the personalized sampling approach, this personalized sampling can only be based on an estimated longitudinal process. If we had compared this personalized sampling approach to the fixed approach in the ‘real’ patient data (based on the unknown natural process), we may have obtained overly optimistic results.

Altogether, our results support the value of maximizing information gain by estimating prognosis in an individual and optimal manner. Because of the strong interaction between kidney function and CHF, such a personalized screening strategy may contribute to efficient monitoring of these complex patients by their treating physicians, and may provide novel opportunities for timely adaptation of treatment. Ultimately, randomized clinical trials should investigate whether personalized screening intervals for kidney biomarkers improve and individualize patient monitoring and herewith improve treatment in patients with CHF.

## Supplementary Information

Below is the link to the electronic supplementary material.

Supplementary file1 (DOCX 2183 KB)

## Data Availability

The data that support the findings of this study will be made available to other researchers for purposes of reproducing the results upon reasonable request and in accordance with a data‐sharing agreement.

## References

[1] Damman K, Valente MAE, Voors AA, O'Connor CM, van Veldhuisen DJ, Hillege HL (2013). Renal impairment, worsening renal function, and outcome in patients with heart failure: an updated meta-analysis. Eur Heart J.

[2] Ponikowski P, Voors AA, Anker SD, Bueno H, Cleland JGF, Coats AJS, Falk V, Gonzalez-Juanatey JR, Harjola VP, Jankowska EA, Jessup M, Linde C, Nihoyannopoulos P, Parissis JT, Pieske B, Riley JP, Rosano GMC, Ruilope LM, Ruschitzka F, Rutten FH, van der Meer P (2016). 2016 ESC Guidelines for the diagnosis and treatment of acute and chronic heart failure: the Task Force for the diagnosis and treatment of acute and chronic heart failure of the European Society of Cardiology (ESC). Eur Heart J.

[3] Brankovic M, Akkerhuis KM, van Boven N, Anroedh S, Constantinescu A, Caliskan K, Manintveld O, Cornel JH, Baart S, Rizopoulos D, Hillege H, Boersma E, Umans V, Kardys I (2018). Patient-specific evolution of renal function in chronic heart failure patients dynamically predicts clinical outcome in the Bio-SHiFT study. Kidney Int.

[4] Brankovic M, Kardys I, Hoorn EJ, Baart S, Boersma E, Rizopoulos D (2018). Personalized dynamic risk assessment in nephrology is a next step in prognostic research. Kidney Int.

[5] Rizopoulos D, Taylor JM, Van Rosmalen J, Steyerberg EW, Takkenberg JJ (2016). Personalized screening intervals for biomarkers using joint models for longitudinal and survival data. Biostatistics.

[6] Schuurman AS, Tomer A, Akkerhuis KM, Brugts JJ, Constantinescu AA, Ramshorst JV, Umans VA, Boersma E, Rizopoulos D, Kardys I (2020) Personalized screening intervals for measurement of *N*-terminal pro-B-type natriuretic peptide improve efficiency of prognostication in patients with chronic heart failure. European journal of preventive cardiology: 2047487320922639. 10.1177/2047487320922639 10.1177/2047487320922639 33611526

[7] van Boven N, Battes LC, Akkerhuis KM, Rizopoulos D, Caliskan K, Anroedh SS, Yassi W, Manintveld OC, Cornel JH, Constantinescu AA, Boersma E, Umans VA, Kardys I (2018). Toward personalized risk assessment in patients with chronic heart failure: detailed temporal patterns of NT-proBNP, troponin T, and CRP in the Bio-SHiFT study. Am Heart J.

[8] Rizopoulos D (2016). The R Package JMbayes for fitting joint models for longitudinal and time-to-event data using MCMC. J Stat Softw.

