# Supplementary material for: Personalized screening intervals for kidney function in patients with chronic heart failure: a modeling study
Source: J Nephrol. 2021 Mar 18;34(5):1421–7. doi: 10.1007/s40620-021-01014-0 (PMC8494722; doi:10.1007/s40620-021-01014-0)
Supplement: Supplementary file 1 — Supplementary file1 (DOCX 2183 KB) [file 40620_2021_1014_MOESM1_ESM.docx]

**Detailed Methods**

*Study design and procedures*

The design of the Bio-SHiFT study has been described in detail elsewhere.[1] Briefly, CHF patients in clinically stable condition were recruited during their regular outpatients visits in the Erasmus MC, Rotterdam, The Netherlands, and Northwest Clinics, Alkmaar, The Netherlands. Patients were eligible if CHF (with reduced or preserved ejection fraction) was diagnosed ≥3 months ago according to the guidelines of the European Society of Cardiology.[2-4] Plasma and urine samples were taken at the day of inclusion and at predefined trimonthly follow-up visits, which were scheduled to a maximum follow-up duration of 30 months. For the current investigation, we used 263 patients who were enrolled during the first inclusion period between October 2011 and June 2013.

During follow-up, occurrence of clinical events was recorded in the electronic case report forms, and associated hospital records and discharge letters were collected. Subsequently, a clinical event committee, blinded to the biomarker-candidate results, reviewed hospital records and discharge letters and adjudicated the study endpoints. The primary study endpoint (PE) was defined as the composite of cardiac death, cardiac transplantation, left ventricular assist device implantation or hospitalization for heart failure, whichever occurred first.

The Bio-SHiFT study was approved by the medical ethics committee of the Erasmus MC and was performed in accordance with the Declaration of Helsinki. Written informed consent was obtained from all patients. The Bio-SHiFT study is registered in ClinicalTrials.gov, number NCT01851538.

*Biomarker measurements*Blood and urine samples were collected at baseline and at each study follow-up visit and were processed and stored at a temperature of –80̊C within 2 hours after collection. Laboratory personnel was blinded to all patient data and findings did not alter patient care. Batch analysis of plasma and urine samples was performed at HaemoScan BV (Groningen, the Netherlands). All urinary biomarkers were normalized to urinary creatinine concentrations to correct for concentration or dilution of urine.

Creatinine was determined by a colorometric test by the Jaffe’s reaction. Plasma was used undiluted, urine was diluted ten times in water (LLD: plasma 0,14 mg/dl, urine: 1.56 mg/ml). CysC was determined in plasma, diluted 2000 times in 0,1%BSA/PBS buffer, by ELISA (R&D systems, Minneapolis, MN) (LLD: 0.1066 µg/mL). KIM-1 was determined in urine, diluted 50% in 0,1% BSA/PBS buffer, by ELISA (R&D systems, Minneapolis, MN, USA) (LLD: 0.146 ng/mL). NAG was determined using a substrate p-nitrophenyl N-acetyl-β-D-glucosaminidase at pH 4.5 (Sigma, St Louis, MO, USA) (LLD: 0.485 U/L).

eGFR was determined by the Chronic Kidney Disease Epidemiology Collaboration (CKD-EPI) creatinine equation as well as the CKD-EPI cystatin C equation.[5,6] Patients were categorized using National Kidney Foundation–Kidney Disease Outcome Quality Initiative (K/DOQI) clinical practice guidelines.[7]

*Statistical analysis*

We utilized a joint model to estimate the association between longitudinally measured biomarker levels and clinical outcome.[8,9] A joint model combines a linear mixed-effect (LME) model for longitudinally measured data with a Cox regression model for time-to-event data. The association between these two types of data is modeled using patient-specific random effects. The LME model uses these random-effects to model the longitudinal temporal pattern of biomarker measurements. The Cox model uses these random-effects to model the impact of the underlying trajectory of biomarker measurements on the risk of PE.[1,10] We used logarithmically (base 2) transformed biomarker measurements in our joint models. Consequently, we were able to obtain a hazard ratio (HR) along with a 95% confidence interval (CI) that estimated the risk of the PE associated with a 20% increase or decrease in biomarker level at a given follow-up time.[1,11]

The potential confounders that we adjusted for in the joint models for kidney markers were age, sex, diabetes, atrial fibrillation, New York Heart Association (NYHA) class, diuretics, systolic blood pressure, and eGFR_creat_ (for tubular markers).[11] Potential confounders adjusted for in the joint model for HsTNT were age, sex, diabetes, NYHA class, body mass index and kidney function.[1] For each biomarker, these potential confounders were used in both the LME and Cox model. Imputations were applied for systolic blood pressure since data on this variable were missing in 5.3% of the patients.

Scheduling personalized screening visits
Scheduling of personalized screening visits is based on the individual patients’ longitudinal biomarker profile.[10] A patient visiting the outpatient clinic has longitudinal biomarker measurements available until a certain time point. From the aforementioned joint model, we can derive for each individual patient the cumulative risk of the PE at a particular follow-up time point, using all of the previously measured biomarker levels until this time point.

For determining the optimal time point for drawing the next sample for biomarker measurement in a particular patient, we first need to establish the cumulative risk of the PE occurring in a certain time window. The time point for drawing the next sample should not be beyond the time point at which the PE occurs. For this reason, we set a maximum limit on the time window based on the cumulative risk of the PE. Then, the time window is defined as the time between the current measurement and the maximum possible time point of drawing the next measurement. We aim to find the optimal time point to draw the next measurement within this time window. We also need to define a risk threshold, which, if crossed within the time window, leads us to stop the further scheduling of measurements, since the patient apparently needs appropriate action and/or increased surveillance, and therefore a different protocol from that point onwards. For this investigation, we have selected a risk threshold of 7.5% for the 3 months that follow, based on clinical considerations. Thus, if the patients’ cumulative risk of the PE exceeds 7.5% within the following 3 months, we stop scheduling further measurements in order to, for example, adjust therapy to avoid the occurrence of the PE. For the current investigation, we focus on the personalized screening schedules themselves, as our primary aim is to enable timely intervention, and to therewith avoid the occurrence of the PE. Hence, for now, we do not propose a specific therapy to be used at the time point that the patients’ cumulative risk of the PE exceeds the risk threshold.

On the other hand, if the cumulative risk of the PE remains less than 7.5% within the following 3 months, we would like to determine the optimal time point at which to obtain the next measurement. The selection of this optimal time point is based on two aspects.[10] First, as stated, the cumulative risk of the PE in the time window should not exceed 7.5%. Second, obtaining a biomarker measurement at this optimal time point should provide us the maximum amount of information about the future cumulative risk of PE for this particular patient. Accordingly, we perform personalized scheduling using the stepwise approach depicted in Fig. 1A-B. Altogether, when applying this approach, patients with relatively stable biomarker profiles will likely not exceed the predefined risk threshold within a specified time window, and the calculations may suggest to wait for a longer time period to perform the next biomarker measurement in these patients. On the other hand, patients with worsening biomarker profiles are more likely to exceed the predefined risk threshold within a specified time window, and the calculations may suggest performing the next biomarker measurement in the short term.

Simulation study

After constructing the joint model and defining the thresholds needed for scheduling personalized screening visits, we proceeded to compare the personalized screening schedule to a fixed screening schedule. For the fixed schedule, we chose trimonthly intervals, in accordance with the design of the Bio-SHiFT study and daily clinical practice. Since our existing data were collected using this fixed screening schedule and hence no ‘real’ data on personalized screening intervals was available, the advantages of a personalized screening design were assessed by means of a simulation study.

We first simulated a dataset containing 263 patients. These 263 simulated patients had baseline characteristics and biomarker profiles similar to the 263 patients included in the Bio-SHiFT, since we simulated using the joint model fitted to the Bio-SHiFT data. First, using the joint model fitted to the Bio-SHiFT data, we generated biomarker measurements at fixed follow-up time points for these 263 simulated patients. This schedule is similar to the schedule of the Bio-SHiFT study. We also generated a true PE time for these patients, as well as a random non-informative censoring time. Subsequently, we fitted a new joint model for these patients and, then, used this model to develop and compare the efficacy of scheduling biomarker measurements according to a fixed screening design and a personalized screening design.

For the personalized screening design, the first three simulated biomarker measurements were considered a given, in order to have a ‘run-in period’ for the patients’ longitudinal biomarker profile, since if we have a longitudinal profile available we can apply the aforementioned stepwise approach of personalized scheduling. Then, in each patient at each follow-up visit, we determined the optimal time point of the next biomarker measurement based on the patient’s individual risk profile as estimated by the joint model and the maximum information gain on the patient’s prognosis as assessed by the Kullback-Leibler divergence, as described by the stepwise approach in Figure 1A-B.[1] We continued planning screening visits until the cumulative risk of the PE exceeded an arbitrary risk threshold of 7.5% within 3-months. Then, we stopped planning to allow for a timely intervention.

The performance of the personalized and fixed screening schedules were compared using two outcome measures; the start of the high-risk interval and the number of scheduled measurements. The high-risk interval was defined as the estimated intervention time minus the true PE time (in months) (Fig. 1C). Thus, the schedule that showed a high-risk interval that was larger in absolute terms (i.e., more negative) was preferred, because such a high-risk interval enables timely intervention. In addition, assuming that the costs of biomarker measurements and outpatient visits remained the same during follow-up, we prefer a procedure that requires the fewest possible repeated measurements. All analyses were performed with R statistical Software using package JMBayes.[12]

**Supplemental results**

*Table S1. Baseline characteristics of patients included in the Bio-SHiFT study.*

| **Table S1. Baseline characteristics** | |
| --- | --- |
| **N = 263 patients** | |
| *Demographical characteristics* | |
| Age, years | 66.7 ± 12.6 |
| Men | 189 (71.9) |
| Caucasian ethnicity | 244 (92.8) |
| *Clinical characteristics* | |
| Body mass index, kg/m^2^ | 27.5 ± 4.7 |
| Heart rate, beat/min | 67.2 ± 11.6 |
| Systolic blood pressure, mmHg | 121.9 ± 20.4 |
| Diastolic blood pressure, mmHg | 72.4 ± 10.9 |
| *Features HF* |  |
| Duration of HF, years | 4.6 (1.7-9.9) |
| NYHA class I or II | 194 (73.8) |
| NYHA class III or IV | 69 (26.2) |
| *Left ventricular function* |  |
| Systolic dysfunction | 250 (95.1) |
| HFPEF | 13 (4.9) |
| LVEF | 32.0 ± 11.7 |
| *Etiology of HF* |  |
| Ischemic heart disease | 117 (44.5) |
| Hypertension | 34 (12.9) |
| Cardiomyopathy | 68 (25.9) |
| Unknown | 19 (7.2) |
| Other | 25 (9.5) |
| *Medical history* |  |
| Myocardial infarction | 96 (36.5) |
| PCI | 82 (31.2) |
| CABG | 43 (16.3) |
| Atrial fibrillation | 106 (40.3) |
| Diabetes mellitus | 81 (30.8) |
| Hypertension | 120 (45.6) |
| *Intoxication* |  |
| Smoking |  |
| Ever | 185 (70.3) |
| Current | 26 (9.9) |
| *Medication use* |  |
| ACE-I or ARB | 245 (93.2) |
| Aldosterone antagonist | 179 (68.1) |
| Diuretic | 237 (90.1) |
| β-Blocker | 236 (89.7) |
| *KDOQI classification* |  |
| *eGFR ≥ 90 ml/min per 1.73m^2^* | *28 (10.6)* |
| *eGFR 60-89 ml/min per 1.73m^2^* | *95 (36.1)* |
| *eGFR 30-59 ml/min per 1.73m^2^* | *118 (44.9)* |
| *eGFR <30 ml/min per 1.73m^2^* | *22 (8.4)* |
| *Biomarker level* |  |
| Creatinine, mg/dl | 1.18 (0.99-1.49) |
| Cystatin C, mg/l | 0.73 (0.57-0.97) |
| NAG, U/gCr | 5.9 (3.8-9.3) |
| KIM-1, ng/gCr | 477.2 (247.0-938.7) |
| HsTNT, ng/l | 18.0 (9.5-33.2) |

Values are mean ± SD, n (%), or median (25^th^-75^th^ percentile). ACE-I, angiotensin-converting enzyme inhibitors; ARB, angiotensin II receptor blockers; CABG, coronary artery bypass grafting; eGFR, estimated glomerular filtration rate; HF, heart failure; HFPEF, heart failure with preserved ejection fraction; LVEF, left ventricular ejection fraction; NYHA, New York Heart Association; PCI, percutaneous coronary intervention.

*Table S2. Baseline characteristics of the original dataset and the simulated dataset as used for the analyses (here the dataset for creatinine is depicted).*

| ***Baseline characteristics*** | ***Original (real) data*** | ***Simulated data*** |
| --- | --- | --- |
| Age | 66.7 ± 12.6 | 67.8 ± 10.7 |
| Men | 189 (71.9) | 187 (71.1) |
| NYHA class I or II | 194 (73.8) | 182 (69.2) |
| NYHA class III or IV | 69 (26.2) | 81 (30.8) |
| Diuretics | 237 (90.1) | 236 (89.7) |
| Diabetes Mellitus | 81 (30.8) | 79 (30.0) |
| Atrial Fibrillation | 106 (40.3) | 115 (43.7) |

*Table S3*

*Similarity of the first three Log2 creatinine and Log2 cystatin c measurements between the real and simulated data.*

|  | **Original data** | **Simulated data** |  |
| --- | --- | --- | --- |
|  | *Median (25^th^-75^th^ percentile) Log2 creatinine value* | *Median (25^th^-75^th^ percentile) Log2 creatinine value* | *p-value* |
| First measurement | 0.24 (-0.01-0.57) | 0.22 (-0.18-0.63) | 0.441 |
| Second measurement | 0.26 (-0.04-0.58) | 0.27 (-0.23-0.75) | 0.893 |
| Third measurement | 0.31 (0.02-0.63) | 0.24 (-0.24-0.80) | 0.442 |

|  | **Original data** | **Simulated data** |  |
| --- | --- | --- | --- |
|  | *Median (25^th^-75^th^ percentile) Log2 cystatin c value* | *Median (25^th^-75^th^ percentile) Log2 cystatin c value* | *p-value* |
| First measurement | -0.45 (-0.82;-0.04) | -0.32 (-0.79-0.10) | 0.090 |
| Second measurement | -0.30 (-0.75-0.06) | -0.35 (-0.78-0.00) | 0.424 |
| Third measurement | -0.37 (-0.74-0.03) | -0.40 (-0.99-0.01) | 0.054 |

*Table S4. Association between temporal biomarker patterns and the primary endpoint in the original data of the Bio-SHiFT study and in simulated datasets.*

| ***Biomarker level*** | ***HR (95% CI) original data*** | ***HR (95% CI) simulated data*** |
| --- | --- | --- |
| Creatinine | 1.12 (1.02-1.23) | 1.15 (1.12-1.18) |
| CystatinC | 2.11 (1.46-3.52) | 1.93 (1.72-2.14) |
| eGFR_creat_ | 1.12 (1.04-1.19) | 1.11 (1.09-1.14) |
| eGFR_cysC_ | 1.14 (1.08-1.21) | 1.14 (1.12-1.16) |
| KIM-1 | 1.06 (1.03-1.09) | 1.07 (1.07-1.08) |
| NAG | 1.07 (1.03-1.12) | 1.07 (1.06-1.09) |

**An illustration reporting the results of two types of screening (fixed and personalized) for an individual patient from a simulated dataset who experienced the PE at 2.46 years of follow-up is depicted in Tables S5 and S6:**

*Figure S1. Individual temporal evolution of creatinine in the example patient.*


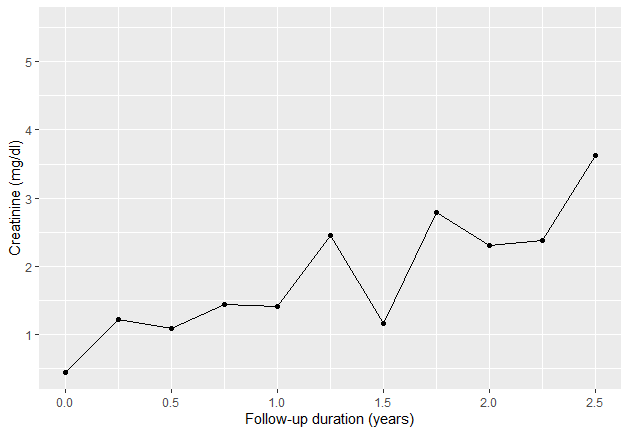


*Table S5. Illustration of predefined, fixed trimonthly scheduling design, starting after ‘run-in period’ of three fixed measurements.*

Fixed trimonthly scheduling is stopped at 2.25 years since at this time point the estimated survival probability exceeds the arbitrarily chosen estimated survival probability of 0.925.

| **Visit number** | **Visit time, years** | **Estimated survival probability** | **Estimated survival probability is <0.925** |
| --- | --- | --- | --- |
| 3 | 0.50 | 0.988 | No |
| 4 | 0.75 | 0.980 | No |
| 5 | 1.00 | 0.975 | No |
| 6 | 1.25 | 0.951 | No |
| 7 | 1.50 | 0.963 | No |
| 8 | 1.75 | 0.939 | No |
| 9 | 2.00 | 0.927 | No |
| 10 | 2.25 | 0.916 | Yes |
| 11 | 2.50 | - | - |

*Table S6. Illustration of personalized scheduling design, starting after ‘run-in period’ of three fixed measurements.*

The visit numbers of the example patient, as performed according to the personalized scheduling design, are depicted in the first column of the table. In the second column the time points of the biomarker measurements are given in years. In the third column the estimated survival probabilities at these time-points are depicted. In the fourth column the time window is defined between the ‘current’ measurement moment, and the time-point at which the cumulative risk of the primary endpoint is estimated at 7.5% (as also illustrated previously in Figure 1). This time window is then subdivided into five, as demonstrated by the five time-points in the fifth column. From these five time-points within this time window, personalized scheduling selects the optimal time-point to perform the next measurement based on the maximum information gain (using the Kullback-Leibler divergence). The lowest numerical value is the most optimal, as demonstrated by the asterisks in the sixth and last column of the table (and as also illustrated in Figure 1).

At visit number three, the personalized approach suggests to wait longer than the fixed approach (1.03 years), as the creatinine value in this patient is relatively low. However, over time, overall, creatinine level increases during follow-up, and for this reason, at visit number four, the personalized screening approach suggests to perform the next, fifth measurement within a shorter time window (0.37 years). The same is true at visit number five at 1.90 years. At this fifth measurement, the time window to select the optimal next, sixth measurement is even further decreased (0.28 years) and the last measurement is performed at 2.07 years. Eventually, personalized scheduling in this patient is stopped at 2.07 years since at this time point the estimated survival probability exceeds the arbitrarily chosen estimated survival probability of 0.925. This individual example illustrates how personalized scheduling appropriately and timely detects the impending event, while using fewer biomarker measurements compared to fixed scheduling.

| **Visit number** | **Visit time, years** | **Estimated survival probability** | **Time window** | **Time points in time window** | **Information gain** | **Selected optimal time point for next measurement** |
| --- | --- | --- | --- | --- | --- | --- |
| 3 | 0.50 | 0.988 | 1.03 | 0.71 | -3.283 |  |
|  |  |  |  | 0.91 | -3.307 |  |
|  |  |  |  | 1.11 | -3.293 |  |
|  |  |  |  | 1.32 | -3.252 |  |
|  |  |  |  | 1.53 | -3.216 | * |
| 4 | 1.53 | 0.950 | 0.37 | 1.60 | -2.020 |  |
|  |  |  |  | 1.67 | -2.065 |  |
|  |  |  |  | 1.75 | -2.039 |  |
|  |  |  |  | 1.82 | -2.052 |  |
|  |  |  |  | 1.90 | -1.979 | * |
| 5 | 1.90 | 0.931 | 0.28 | 1.95 | -1.549 |  |
|  |  |  |  | 2.01 | -1.585 |  |
|  |  |  |  | 2.07 | -1.535 | * |
|  |  |  |  | 2.12 | -1.578 |  |
|  |  |  |  | 2.18 | -1.556 |  |
| 6 | 2.07 | 0.891 | - | - | - | - |

** Denotes the optimal time point within a time window to perform the next measurement, as selected by the personalized screening approach.*

*HsTNT*Personalized scheduling of HsTNT measurements, as compared to fixed scheduling, demonstrated similar prognostic performance but required fewer measurements (personalized; median of 6 (25^th^-75^th^ percentile:5-7) and fixed; median of 12 (25^th^-75^th^ percentile:9-13)) (Figure S2), resulting in a median (25^th^-75^th^ percentile) of 1.6 (0.7-2.3) saved measurements per year.

*Figure S2. Comparison of personalized and fixed scheduling of HsTNT.*


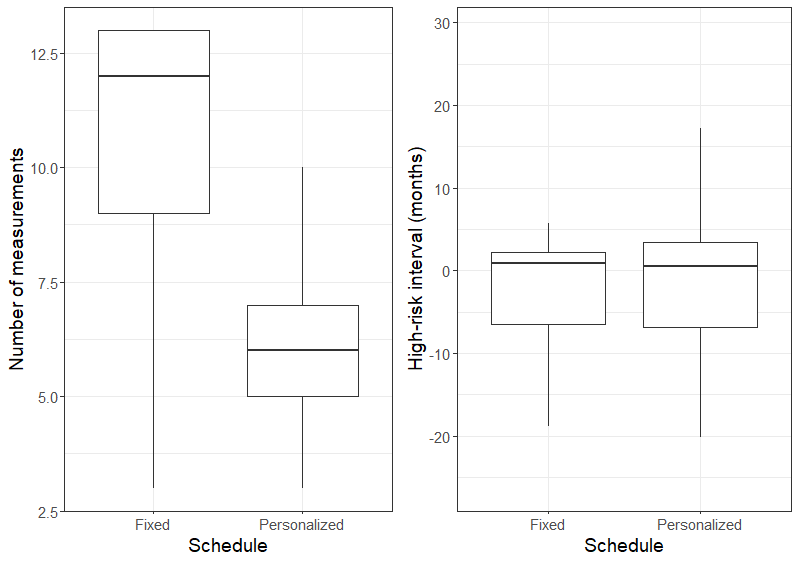


**References**

1. van Boven N, Battes LC, Akkerhuis KM, Rizopoulos D, Caliskan K, Anroedh SS, Yassi W, Manintveld OC, Cornel JH, Constantinescu AA, Boersma E, Umans VA, Kardys I (2018) Toward personalized risk assessment in patients with chronic heart failure: Detailed temporal patterns of NT-proBNP, troponin T, and CRP in the Bio-SHiFT study. American heart journal 196:36-48. doi:10.1016/j.ahj.2017.10.008

2. McMurray JJ, Adamopoulos S, Anker SD, Auricchio A, Böhm M, Dickstein K, Falk V, Filippatos G, Fonseca C, Gomez-Sanchez MA, Jaarsma T, Køber L, Lip GY, Maggioni AP, Parkhomenko A, Pieske BM, Popescu BA, Rønnevik PK, Rutten FH, Schwitter J, Seferovic P, Stepinska J, Trindade PT, Voors AA, Zannad F, Zeiher A, Guidelines ESCCfP (2012) ESC Guidelines for the diagnosis and treatment of acute and chronic heart failure 2012: The Task Force for the Diagnosis and Treatment of Acute and Chronic Heart Failure 2012 of the European Society of Cardiology. Developed in collaboration with the Heart Failure Association (HFA) of the ESC. European heart journal 33 (14):1787-1847. doi:10.1093/eurheartj/ehs104

3. Paulus WJ, Tschöpe C, Sanderson JE, Rusconi C, Flachskampf FA, Rademakers FE, Marino P, Smiseth OA, De Keulenaer G, Leite-Moreira AF, Borbély A, Édes I, Handoko ML, Heymans S, Pezzali N, Pieske B, Dickstein K, Fraser AG, Brutsaert DL (2007) How to diagnose diastolic heart failure: a consensus statement on the diagnosis of heart failure with normal left ventricular ejection fraction by the Heart Failure and Echocardiography Associations of the European Society of Cardiology. European Heart Journal 28 (20):2539-2550

4. Dickstein K, Cohen-Solal A, Filippatos G, McMurray JJV, Ponikowski P, Poole-Wilson PA, Strömberg A, Van Veldhuisen DJ, Atar D, Hoes AW, Keren A, Mebazaa A, Nieminen M, Priori SG, Swedberg K, Vahanian A, Camm J, De Caterina R, Dean V, Funck-Brentano C, Hellemans I, Kristensen SD, McGregor K, Sechtem U, Silber S, Tendera M, Widimsky P, Zamorano JL, Auricchio A, Bax J, Böhm M, Corrà U, Della Bella P, Elliott PM, Follath F, Gheorghiade M, Hasin Y, Hernborg A, Jaarsma T, Komajda M, Kornowski R, Piepoli M, Prendergast B, Tavazzi L, Vachiery JL, Verheugt FWA, Zannad F (2008) ESC Guidelines for the diagnosis and treatment of acute and chronic heart failure 2008. European Heart Journal 29 (19):2388-2442. doi:10.1093/eurheartj/ehn309

5. McAlister FA, Ezekowitz J, Tarantini L, Squire I, Komajda M, Bayes-Genis A, Gotsman I, Whalley G, Earle N, Poppe KK, Doughty RN (2012) Renal dysfunction in patients with heart failure with preserved versus reduced ejection fraction: impact of the new Chronic Kidney Disease-Epidemiology Collaboration Group formula. Circulation Heart failure 5 (3):309-314. doi:10.1161/circheartfailure.111.966242

6. Inker LA, Schmid CH, Tighiouart H, Eckfeldt JH, Feldman HI, Greene T, Kusek JW, Manzi J, Van Lente F, Zhang YL, Coresh J, Levey AS (2012) Estimating glomerular filtration rate from serum creatinine and cystatin C. The New England journal of medicine 367 (1):20-29. doi:10.1056/NEJMoa1114248

7. K/DOQI clinical practice guidelines for chronic kidney disease: evaluation, classification, and stratification (2002). American journal of kidney diseases : the official journal of the National Kidney Foundation 39 (2 Suppl 1):S1-266

8. Rizopoulos D (2012) Joint Models for Longitudinal and Time-to-Event Data: With Applications in R. Taylor & Francis,

9. Tsiatis AA, Davidian M (2004) Joint modeling of longitudinal and time-to-event data: an overview. Statistica Sinica 14 (3):809-834

10. Rizopoulos D, Taylor JM, Van Rosmalen J, Steyerberg EW, Takkenberg JJ (2016) Personalized screening intervals for biomarkers using joint models for longitudinal and survival data. Biostatistics 17 (1):149-164

11. Brankovic M, Akkerhuis KM, van Boven N, Anroedh S, Constantinescu A, Caliskan K, Manintveld O, Cornel JH, Baart S, Rizopoulos D, Hillege H, Boersma E, Umans V, Kardys I (2018) Patient-specific evolution of renal function in chronic heart failure patients dynamically predicts clinical outcome in the Bio-SHiFT study. Kidney international 93 (4):952-960. doi:10.1016/j.kint.2017.09.013

12. Rizopoulos D (2016) The R Package JMbayes for Fitting Joint Models for Longitudinal and Time-to-Event Data Using MCMC. Journal of Statistical Software 72 (7):46 . doi:10.18637/jss.v072.i07
